# Type IV fimbrial subunit protein ApfA contributes to protection against porcine pleuropneumonia

**DOI:** 10.1186/1297-9716-43-2

**Published:** 2012-01-12

**Authors:** Lenka Sadilkova, Jiri Nepereny, Vladimir Vrzal, Peter Sebo, Radim Osicka

**Affiliations:** 1 Institute of Microbiology of the Academy of Sciences of the Czech Republic, v.v.i., Videnska 1083, CZ-142 20 Prague, Czech Republic; 2 Bioveta, a. s., Komenskeho 212/12, CZ-683 23 Ivanovice na Hane, Czech Republic

## Abstract

Porcine pleuropneumonia caused by *Actinobacillus pleuropneumoniae *accounts for serious economic losses in the pig farming industry worldwide. We examined here the immunogenicity and protective efficacy of the recombinant type IV fimbrial subunit protein ApfA as a single antigen vaccine against pleuropneumonia, or as a component of a multi-antigen preparation comprising five other recombinant antigens derived from key virulence factors of *A. pleuropneumoniae *(ApxIA, ApxIIA, ApxIIIA, ApxIVA and TbpB). Immunization of pigs with recombinant ApfA alone induced high levels of specific serum antibodies and provided partial protection against challenge with the heterologous *A. pleuropneumoniae *serotype 9 strain. This protection was higher than that engendered by vaccination with rApxIVA or rTbpB alone and similar to that observed after immunization with the tri-antigen combination of rApxIA, rApxIIA and rApxIIIA. In addition, rApfA improved the vaccination potential of the penta-antigen mixture of rApxIA, rApxIIA, rApxIIIA, rApxIVA and rTbpB proteins, where the hexa-antigen vaccine containing rApfA conferred a high level of protection on pigs against the disease. Moreover, when rApfA was used for vaccination alone or in combination with other antigens, such immunization reduced the number of pigs colonized with the challenge strain. These results indicate that ApfA could be a valuable component of an efficient subunit vaccine for the prevention of porcine pleuropneumonia.

## Introduction

*Actinobacillus pleuropneumoniae*, the etiological agent of porcine pleuropneumonia, is a Gram-negative bacterium colonizing the porcine respiratory tract [1-3]. Pleuropneumonia is a severe contagious and economically significant disease. It can range from acute to chronic, depending on host age, immune status, the bacterial strain causing the infection, or the infective dose [4-6]. The acute stage is characterized by a haemorrhagic necrotizing pneumonia and fibrinous pleuritis and may progress rapidly to death [7,8]. In the chronic stage, localized lung lesions and adhesive pleuritis can be observed and chronically infected animals can become a source of infection for the whole non-infected herd [1,2,9].

To control porcine pleuropneumonia, vaccination is useful [10,11], but development of efficient vaccines against the disease appears difficult due to the existence and diversity of 15 serotypes of *A. pleuropneumoniae *that are differentiated on the basis of surface polysaccharide antigens [12-14]. The first vaccines against *A. pleuropneumoniae *infection comprised formalin-treated or heat-inactivated bacteria. These whole-cell bacterin vaccines reduce mortality after challenge with the homologous serotypes of *A. pleuropneumoniae*, but usually do not confer efficient protection against infection with heterologous serotypes [15-17]. The limited protection observed with bacterins might be explained by (i) the absence of secreted immunogenic proteins, such as the ApxA toxins that are the key virulence factors of *A. pleuropneumoniae*, (ii) the alteration of antigenic potency of certain bacterial antigens due to inactivation treatment, or (iii) the absence of immunogenic antigens that are expressed only within the host [11,18-22]. Indeed, pigs surviving natural or experimental infection with *A. pleuropneumoniae *were found to be completely protected against homologous serotypes and generally also against heterologous serotype infections [16,23,24]. To overcome the drawbacks of bacterins, live attenuated vaccines that reflect natural *A. pleuropneumoniae *infection and allow the in-vivo production of immunogenic antigens were developed, comprising temperature-sensitive, streptomycin-dependent or metabolic mutants, or mutants having deleted or inactivated genes for key virulence factors [25-34]. Some of the live attenuated vaccines tested indeed confer a high-level cross-protection in contrast to whole-cell bacterin vaccines [30,35,36]. Despite many promising results, the use of live bacteria brings numerous safety drawbacks that could be eliminated by the development of a highly efficient subunit vaccine. Among the valuable components of different subunit vaccines, the key virulence factors of *A. pleuropneumoniae*, such as the ApxA exotoxins, the outer membrane proteins, or iron-acquisition factors, were tested, respectively [11].

*A. pleuropneumoniae *secretes three different ApxA exotoxins (ApxIA, ApxIIA, and ApxIIIA), which are members of the RTX (Repeat in ToXin) family [3,37-42]. ApxIA shows strong hemolytic activity, while ApxIIA exhibits weaker hemolytic activity [43,44]. Both are cytotoxic and active on a broad range of cells of different types and species [45]. ApxIIIA is nonhemolytic, but it is strongly cytotoxic and targets mainly porcine alveolar macrophages and neutrophils [44,46]. The ApxA exotoxins are thought to be of particular importance as antigens for inducing protective immunity against pleuropneumonia and have been included in a broad range of tested subunit vaccines [21,47-49]. A fourth secreted RTX protein of *A. pleuropneumoniae*, ApxIVA, was described and its biologic activity remains unknown [50]. ApxIVA appears to be produced in vivo but not under in vitro conditions [19,50]. Recently, the contribution of recombinant ApxIVA to the protective efficacy of a subunit vaccine against *A. pleuropneumoniae *challenge was demonstrated [49].

To survive in the iron-depleted environment of the host, *A. pleuropneumoniae *produces the surface proteins, TbpA and TbpB (transferrin binding proteins), that enable the pathogen to utilize porcine transferrin as a source of iron [51]. Both transferrin binding proteins are key virulence factors of *A. pleuropneumoniae *[52]. It has been shown that pigs immunized with recombinant TbpB are less susceptible to the disease after experimental homologous challenge with *A. pleuropneumoniae *serotype 7 [53].

Since each of the *A. pleuropneumoniae *proteins alone offers only a partial protection against porcine pleuropneumonia, current commercially available or newly tested vaccines consist of combinations of several bacterial antigens [21,47-49,53]. These subunit vaccines are effective at preventing acute disease, but do not sufficiently protect against colonization and are not widely cross-protective [33,47]. Therefore, further *A. pleuropneumoniae *outer membrane and/or secreted proteins are screened and tested as candidates for inclusion into next generation vaccines [54]. Among the promising antigens to be included in a subunit vaccine against porcine pleuropneumonia is the type IV fimbrial subunit protein. Type IV fimbriae (pili) are typically composed of thousands of subunit proteins polymerized into a fiber that protrudes from the bacterial cell surface [55]. Fimbriae are, indeed, known to play an important role in the pathogenesis of Gram-negative bacteria such as *Moraxella catarrhalis, Neisseria meningitidis, N. gonorrhoeae, Haemophilus influenzae*, enteropathogenic *Escherichia coli *or *Pseudomonas aeruginosa *and are involved in adherence and colonization, twitching motility, biofilm formation, protein export and DNA uptake [56-63]. Type IV fimbriae were also isolated from *A. pleuropneumoniae *and a 15.9-kDa ApfA protein was identified as the type IV fimbrial subunit protein [55]. The *apfA *gene was found to be present in 12 representative *A. pleuropneumoniae *serotype strains and exhibited greater than 98% identity on DNA level with the predicted amino acid sequences being 100% identical [64]. Moreover, all typical fimbrial biogenesis genes were identified in *A. pleuropneumoniae *and appear to form a type IV fimbrial operon with *apfA *[18]. This is present in a single copy in the *A. pleuropneumoniae *genome and was found to be expressed in vivo, but not in vitro [18]. The single copy and high conservation of the *apfA *gene, the surface location of the ApfA protein and its putative role in adherence, colonization and other important biological functions, make ApfA a particularly attractive target for vaccine development.

Here, we tested the immunogenicity and protective potential of recombinant ApfA against porcine pleuropneumonia, either in a single antigen vaccine, or as a component of a multi-antigen preparation, including five other purified recombinant antigens derived from key virulence factors of *A. pleuropneumoniae*.

## Materials and methods

### Bacterial strains and growth conditions

The *E. coli *K12 strain, XL1-Blue (Stratagene, La Jolla, USA), was used throughout this work for DNA manipulations and was grown in Luria-Bertani (LB) medium supplemented with 60 μg/mL of kanamycin. The *E. coli *BL21(λDE3) strain (Invitrogene, Carlsbad, USA) was used for expression of recombinant proteins and was grown at 37°C in LB medium containing 60 μg/mL of kanamycin.

*A. pleuropneumoniae *serotypes 1, 2, 3 and 7 were used for isolation of genomic DNA and *A. pleuropneumoniae *serotype 9 strain was used for challenge experiments. *A. pleuropneumoniae *serotype 7 strain was obtained from the Veterinary Research Institute, Czech Republic - Collection of animal pathogenic microorganisms (CAPM 3800 serovar 7, strain: WF 83, S. Rosendal, Canada, source: pig/lung (pleuropneumonia)). All other serotypes of *A. pleuropneumoniae *were isolated in the Moravske Prusy agricultural co-operative (Czech Republic) from the lungs of pigs with respiratory infection (after necropsy). It was verified by PCR-cloning and DNA sequencing that the serotype 9 strain used for challenge experiments harbored a fully conserved *apfA *gene allele.

*A. pleuropneumoniae *was grown on Columbia agar base (Lab M, Bury, UK) supplemented with 10% NAD (Sigma-Aldrich, St. Louis, USA) for 24 h at 37°C in 5% CO_2 _and then inoculated into the liquid culture (Supplemented Columbia broth (BBL Microbiology Systems, Cockeysville, USA)) supplemented with 1% IsoVitaleX (BBL Microbiology Systems, Cockeysville, USA) and 10 μg/mL of NAD, grown for 6 h and harvested by centrifugation at 4 500 × *g *for 15 min.

### Isolation and cloning of genes encoding ApfA, ApxA and TbpB proteins

The *A. pleuropneumoniae *genomic DNA was isolated from serotypes 1, 2, 3 and 7 using phenol-chloroform extraction [65] and the *apfA, apxIA, apxIIA, apxIIIA, apxIVA *and *tbpB *genes were amplified by polymerase chain reaction (PCR) (Table 1). Reaction mixtures (70 μL) contained 100 ng of genomic DNA, 2 μM of each oligonucleotide primer, 100 mM KCl, 20 mM Tris-HCl (pH 8.3), 7.5 mM MgCl_2_, 1 mM DTT, 200 μg/mL BSA, 140 μM of each dNTP and 1 unit of Phusion DNA polymerase (Finnzymes, Espoo, Finland). Reaction mixtures were pre-heated to 98°C for 30 s and subjected to 30 cycles of 98°C for 10 s, 52°C for 30 s, and 72°C for 3 min using a PCR Sprint instrument (ThermoHybaid, Middlesex, UK). Specific primer pairs for PCR amplification were designed based on the published sequences of the *apfA, apxIA, apxIIA, apxIIIA, apxIVA *and *tbpB *genes (Table 1) [37,66,67]. To facilitate cloning, the primers were designed to produce a PCR product with a restriction site for the *Sal *I enzyme at the 5'-end and for *Not *I at the 3'-end, respectively (Table 1). PCR products were isolated from agarose gel, digested with the *Sal *I and *Not *I restriction enzymes and fused in frame with the sequence for 6 × His tag at the 5'- and 3'-ends by cloning into the *Sal *I and *Not *I sites of the modified pET28b_*NcoSal *vector. This construct was prepared by ligation of *Nco *I - *Sal *I digested pET28b (Novagen, Madison, USA) with a double-stranded adapter (5'-CATGCATCATCATCATCATCATCATAG-3' and 5'-TCGACTATGATGATGATGATGATGATG-3'). The set of expression vectors, pET28b-*apfA*, pET28b-*apxIA*, pET28b-*apxIIA*, pET28b-*apxIIIA*, pET28b-*apxIVA *and pET28b-*tbpB*, was verified by restriction analysis and DNA sequencing.

**Table 1:** PCR primer pairs, origin and features of *apfA, apxA *and *tbpB *genes and corresponding proteins

Gene^1^	*A. pleuropneumoniae * serotype	PCR primer pair (5' → 3')^2^	Gene length (bps)	Protein MW (kDa)^3^
***apfA***	7	CC**GTCGAC**ATGTCATATAACAGTTATAC CC**GCGGCCGC**ATTTGATGCGCAGAAATTT	342	14
***apxIA***	1	GG**GTCGAC**ACGCTAACTCTCAGCTCGATA GG**GCGGCCGC**AGCTGCTTGTGCTAAAGAA	3069	113
***apxIIA***	7	CC**GTCGAC**ACTCAAAAATCACTTTGTCATCA CC**GCGGCCGC**AGCGGCTCTAGCTAATTG	2871	105
***apxIIIA***	2	GG**GTCGAC**ACAGTACTTGGTCAAGCATGTT GG**GCGGCCGC**AGCTGCTCTAGCTAGGTTA	3159	115
***apxIVA***	3	CC**GTCGAC**ACACAAAATTAACTATGCAAGA CC**GCGGCCGC**TAAAGCAGCTGTTAAGCTATT	5418	205
***tbpB***	7	CC**GTCGAC**ACTCTGGCGGAAAAGGAAGTT CC**GCGGCCGC**TTTTTTTACTTGTTGTTTTGCA	1644	60

^1^Sequences of genes were acquired from GenBank (*apfA*, AY235719.1; *apxIA*, AF240779; *apxIIA*, CP001091; *apxIIIA*, L12145; *apxIVA*, AF030511; *tbpB*, U16017).

^2^All oligonucleotides were synthesized by Generi Biotech, Hradec Kralove, Czech Republic. First oligonucleotide represents forward PCR primer, second reverse PCR primer. *Sal *I and *Not *I recognition sites are marked in bold and underlined.

^3^The approximate protein molecular weight was calculated using the Compute pI/Mw tool (ExPASy).

### Protein production and purification

All recombinant proteins were produced in *E. coli *strain BL21(λDE3) transformed with the appropriate plasmid. Exponential 500 mL cultures grown with shaking at 37°C in LB medium supplemented with 60 μg/mL of kanamycin were induced at OD_600 _= 0.6 - 0.8 with 1 mM IPTG and grown for an additional 4 h. The cells were harvested by centrifugation, washed twice with 50 mM Tris-HCl (pH 8.0), 150 mM NaCl (TN buffer) and disrupted by sonication at 4°C. The extracts were cleared at 20 000 × *g *for 20 min, supernatants were discarded and pellets were solubilized with 50 mM Tris-HCl (pH 8.0), 8 M urea and 300 mM NaCl (TUN buffer) and centrifuged at 20 000 × *g *for additional 20 min. The obtained urea extracts were loaded on a Ni-NTA agarose column (Qiagen, Valencia, USA) equilibrated with the TUN buffer. Washing was performed with equilibration buffer and recombinant proteins were eluted with TUN buffer supplemented with 250 mM imidazole. To remove the imidazole from the samples, the most concentrated fractions were loaded on Sephadex G-25 (Sigma-Aldrich, St. Louis, USA) and transferred into the TUN buffer.

### DNA manipulations and recombinant protein analysis

SDS polyacrylamide gel electrophoresis (SDS-PAGE), Western blot analysis, determination of protein concentrations and in vitro DNA manipulations were performed according to standard protocols [65].

### Preparation of A. pleuropneumoniae for challenge experiments

*A. pleuropneumoniae *serotype 9 strain was grown to OD_600 _= 0.6, culture was centrifuged at 4 500 × *g *for 15 min and resuspended in sterile PBS (phosphate-buffered saline, pH 7.4). The suspension was plated in triplicate in 10-fold serial dilutions onto Columbia agar supplemented with 10% NAD and grown for 24 h at 37°C in 5% CO_2 _for the exact estimation of the number of colony-forming units (CFU). The bacteria used for challenge experiments were tested by Western blot for the production of ApxIA and ApxIIA toxins that are known to be of major importance for the full virulence of *A. pleuropneumoniae *. The challenge suspension was administered to experimental animals using an intranasal probe.

### Experimental animals

For the immunization and challenge experiments, 8-week old piglets (Agricultural Co-operative, Tesnovice, Czech Republic) of both genders, cross-breeds of Czech White and Landrace, weighting 21-24 kg, were used. All piglets were tested prior to vaccination for the absence of serum antibodies against *A. pleuropneumoniae *(Chekit APP-ApxIV, IDEXX Laboratories, Westbrook, USA), *Pasteurella multocida *(PMT ELISA Kit, DakoCytomation, Glostrup, Denmark), *Mycoplasma hyopneumoniae *(HerdChek *M. hyo*, IDEXX), *Salmonella *spp. (HerdChek Swine *Salmonella*, IDEXX), porcine reproductive and respiratory syndrome virus (IDEXX PRRS X3, IDEXX), swine influenza virus (CIVTEST Suis Influenza, Hipra Laboratories, Gerona, Spain) and pseudorabies virus (HerdChek Anti-PRV gB, IDEXX), respectively. None of the piglets showed disease signs before or at the time of challenge. Handling with animals was performed according to the national legislation on animal welfare and in line with internal guidelines of Bioveta, a. s. (SOPs of Bioveta and the Guidelines for the Care and Use of Laboratory Animals, the Act of the Czech National Assembly, Collection of laws No. 149/2004, inclusive of the amendments, on the Protection of Animals against Cruelty, and Public Notice of the Ministry of Agriculture of the Czech Republic, Collection of laws No. 207/2004, on keeping and exploitation of experimental animals).

### Determination of *A. pleuropneumoniae *challenge dose in pigs

The challenge dose for the intranasal infection, eliciting overt signs of severe porcine pleuropneumonia and causing mortality in 50% of animals (LD50), was determined for a local *A. pleuropneumoniae *serotype 9 strain. Three groups of four pigs were inoculated intranasally with 3 × 10^6^, 1 × 10^7 ^and 2 × 10^8 ^CFU (each 5 mL dose was split into two 2.5 mL aliquots and applied to both nostrils) of bacteria and the pigs were monitored daily for 4 days for signs of morbidity and mortality rate. Two out of four challenged pigs died within 24 hours from challenge with the dose of 2 × 10^8 ^CFU, which was used further as the challenge dose in tests of vaccine potency.

### Immunizations and challenge experiments in pigs

The immunization and challenge experiments were performed twice and the results obtained exhibited a good concordance. In total, twenty-five pigs in experiment 1 and nineteen pigs in experiment 2, respectively, were randomly allocated into 8 groups (Table 2) and sampled to obtain preimmune sera. Animals in groups 1 to 6 were intramuscularly injected with purified recombinant antigens in oil adjuvant (antigen(s) diluted in 1.5 mL of PBS to a final amount indicated in the text below plus 0.5 mL of EMULSIGEN, MVP laboratories, Inc., USA) as follows: Group 1) 12 pigs, immunized with rApfA (100 μg per dose); group 2) 3 pigs, injected with rApxIVA (100 μg per dose); group 3) 3 pigs, immunized with rTbpB (100 μg per dose); group 4) 3 pigs, immunized with a combination of rApxIA, rApxIIA and rApxIIIA (50 μg each per dose); group 5) 7 pigs, injected with a penta-antigen combination of rApxIA, rApxIIA, rApxIIIA, rApxIVA and rTbpB proteins (50 μg each per dose); group 6) 7 pigs, immunized with a hexa-antigen vaccine including rApfA (100 μg per dose) and rApxIA, rApxIIA, rApxIIIA, rApxIVA, rTbpB (50 μg each per dose). Two weeks later all the groups were revaccinated with the same dose of antigens. Seven pigs in group 7 received PBS in oil adjuvant and 2 pigs in group 8 were left non-vaccinated. The post-vaccination sera from all piglets were collected one day before challenge and the levels of antibodies against the antigens were tested by ELISA. Animals in groups 1 to 7 were intranasally challenged one month after the first immunization. Inocula were applied using an intranasal probe and consisted of the LD_50 _infectious dose of *A. pleuropneumoniae *serotype 9 strain (2 × 10^8^CFU in 5 mL). Following challenge, piglets were examined and scored daily over seven days using a 0 to 4 scale: 0, no signs of porcine pleuropneumonia; 1, increased respiration rate, and/or sporadic coughing, and/or occasional lying down, and/or mild apathy; 2, abdominal respiration, and/or coughing, and/or lying down, and/or apathy; 3, dyspnea, and/or regular coughing, and/or lying down, and/or markedly depressed appearance, and/or bloody discharge, and/or agony; 4, death. Moreover, body temperature was taken daily. On day 7 post-challenge all surviving pigs were sacrificed and their lungs were subjected to examination for lesions. These were scored as previously described [49,68] with the following modifications: 0, no pathological changes; 1, minor pathological changes, 1-20% of the lobe affected; 2, larger damage of the lobe, 21-50% of the lobe affected; 3, severe damage of the lobe, 51-100% of the lobe affected. The left and right cranial, middle and caudal lobes were scored separately and the mean lung lesion score was calculated for each animal. To check for presence of the challenge bacteria, the cranial lobe of each lung and lung portions exhibiting lesions were replica printed on a blood agar plate (Columbia Blood Agar Base, HIMEDIA, Mumbai, India) containing 6% of sheep defibrinated blood. Colonies of *A. pleuropneumoniae *were grown for 48 h at 37°C in the hemolytic zone of *Staphylococcus aureus *Cowan I (CCM 2352; Veterinary Research Institute, Brno, Czech Republic). After incubation, the presence of satellite colonies was scored as suspect positive for *A. pleuropneumoniae *. Suspect colonies were confirmed as *A. pleuropneumoniae *serotype 9 strain by the PCR method and the coagulation test as previously described [69,70].

**Table 2:** Clinical score, lung lesion score and re-isolation of the challenge strain after immunization of pigs with different vaccines, followed by heterologous intranasal challenge with *A. pleuropneumoniae *

Vaccine^1^	Number of pigs^2^	Clinical score^3^	Body temperature (°C)^4^	Lung lesion score^5^	Re-isolation of the challenge strain (%)^6^	Surviving pigs (%)
rApfA	12	1.7 ± 0.7^7^	40.3 ± 0.5^7^	1.3 ± 0.7^7^	58^7^	100
rApxIVA	3	2.6 ± 0.5	40.8 ± 0.4	1.8 ± 0.5	100	100
rTbpB	3	2.7 ± 0.5	40.9 ± 0.5	1.9 ± 0.7	100	100
rApxIA+rApxIIA+rApxIIIA	3	1.9 ± 0.7	40.5 ± 0.4	1.5 ± 0.6	100	100
rApxIA+rApxIIA+rApxIIIA+rApxIVA+rTbpB	7	1.2 ± 0.5^7^	39.9 ± 0.4^7^	1.0 ± 0.6^7^	86	100
rApfA+rApxIA+rApxIIA+rApxIIIA+rApxIVA+rTbpB	7	0.3 ± 0.5^7,8^	39.6 ± 0.5^7^	0.3 ± 0.5^7,8^	29^7^	100
Control (PBS in oil adjuvant)	7	3.2 ± 0.7	41.2 ± 0.4	2.3 ± 0.7	100	57
Non-treated	2	0	39.1 ± 0.4	0	0	100

^1^Pigs were immunized with two doses of the indicated vaccines at two week intervals and challenged intranasally two weeks later with *A. pleuropneumoniae *serotype 9 strain (2 × 10^8^CFU).

^2^Two independent immunization and challenge experiments were performed. Total number of pigs per vaccine group is given.

^3^Following the challenge, pigs were examined for clinical signs of pleuropneumonia and scored every day for seven days using a 0 to 4 scale. Mean value for each vaccination group calculated from two independent challenge experiments ± standard deviation is given.

^4^Following the challenge, body temperature was taken daily for seven days. Mean value for each vaccination group calculated from two independent challenge experiments ± standard deviation is given.

^5^Upon death, or immediately upon sacrifice on day seven, all animals underwent necropsy and macroscopic inspection of lungs. The observed lung lesions were scored using a 0 to 3 scale and mean value for each group calculated from two independent challenge experiments ± standard deviation is given.

^6^Percentages of pigs in each vaccination group from which the challenge strain of A. p*leuropneumoniae *was re-isolated.

^7^Statistically significant difference (*P *< 0.02) between the vaccine immunized group and the control group, each comprising at least 7 animals.

^8^Statistically significant difference (*P *< 0.02) between the combined hexa-antigen vaccine (rApfA, rApxIA to IVA and rTbpB) immunized group and the penta-antigen vaccine (rApxIA to IVA and rTbpB) immunized group.

### ELISA

Wells of the PolySorp ELISA plates (Nunc, Roskilde, Denmark) were coated with 100 μL per well of particular purified protein antigen in concentration of 32 ng/mL (rApfA), 63 ng/mL (rApxIA), 63 ng/mL (rApxIIA), 32 ng/mL (rApxIIIA), 32 ng/mL (rApxIVA) and 750 ng/mL (rTbpB), respectively. In addition, a mock extract of *E. coli *BL21(λDE3), processed by the same chromatographic procedure as the recombinant proteins, was also used for coating, in order to control the reaction of sera to any residual *E. coli *components within the protein preparations. Plates were coated overnight at 4°C, washed three times with 300 μL of PBS containing 0.05% Tween-20 (Sigma-Aldrich, St. Louis, USA) (PBST) and blocked for 1 h with 1% BSA (Sigma-Aldrich, St. Louis, USA) in PBST. Serial dilutions of individual hyperimmune sera were added and the plates were incubated for 1 h at 37°C. After rinsing with PBST, peroxidase-conjugated rabbit anti-swine IgG (Amersham Biosciences, Piscataway, USA) was used at a 1:3 000 dilution and the plates were incubated for 1 h at 37°C. After washing with PBST and incubation with o-phenylenediamine peroxidase substrate solution, the reactions were stopped with 2 M sulphuric acid and read at 492 nm using a Safire II microplate reader (Tecan, Zürich, Switzerland). The cutoff values for determination of anti-antigen (rApfA, rApxIA, rApxIIA, rApxIIIA, rApxIVA or rTbpB) IgG antibody titers were determined as the mean plus two standard deviations from the test results of preimmune sera at 1:100 dilution. The titers of antigen-specific antibodies were then defined as the reciprocal of the last dilution yielding an absorbance at 492 nm greater than the cutoff value.

### Statistical analysis

The values of clinical signs, body temperatures, lung lesion scores and antibody titers were calculated from the data obtained for particular animals included in a given vaccination group and the numbers in the text, figures and tables thus represent mean values ± standard deviations. To analyze the differences in various parameters among the vaccination groups, the Student's *t *-test was used on groups comprising at least 7 animals and the threshold for significance was set as *P *< 0.02.

## Results

### Cloning, expression and purification of recombinant antigens

**Figure 1:**
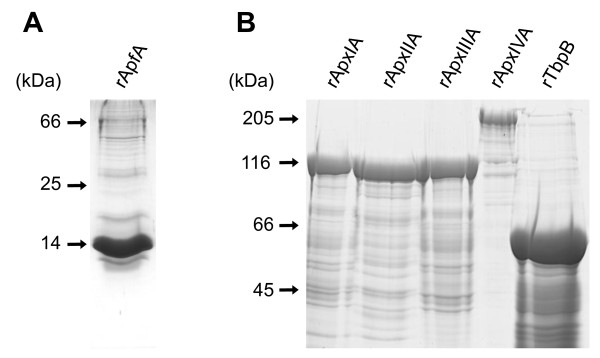
**Purified recombinant ApfA, ApxIA, ApxIIA, ApxIIIA, ApxIVA and TbpB proteins**. Recombinant proteins were purified on Ni-NTA agarose columns, separated on a 15% (rApfA) (A) or 7.5% (all other proteins) (B) polyacrylamide gel and stained with Coomassie Blue.

The genes coding for ApfA, ApxIA, ApxIIA, ApxIIIA, ApxIVA and TbpB were amplified from genomic DNA of *A. pleuropneumoniae *strains, cloned into an expression vector and overexpressed in *E. coli *cells. Since the production of the full-length ApfA protein was shown to be highly toxic for *E. coli *[64], ApfA lacking the N-terminal secretion signal and a predicted hydrophobic region (residues 1 to 35) was produced. The removal of the hydrophobic portion was expected to prevent the potential aggregation of the recombinant ApfA and the observed toxicity to *E. coli *cells [64]. All recombinant antigens were purified close to homogeneity from inclusion bodies by an affinity chromatography on Ni-NTA agarose. This procedure yielded per liter of bacterial culture approximately 5 mg of rApfA, 40 mg of rApxIA, rApxIIA or rApxIIIA, 15 mg of rApxIVA and 50 mg of rTbpB, respectively. Homogeneity of the final protein preparations was tested by SDS-PAGE (Figure 1) and the identity of the recombinant antigens was confirmed by MALDI-TOF mass spectrometry (data not shown).

### Immunization with the ApfA antigen reduces clinical signs of porcine pleuropneumonia and significantly increases the potency of a multi-antigen vaccine

Prior to challenge, the pigs were randomly allocated into 8 groups and intramuscular immunization was performed with rApfA, rApxIVA or rTbpB individually, or with a tri-antigen combination of rApxIA to IIIA, or with a combined penta-antigen vaccine containing rApxIA to IVA and rTbpB, or with a hexa-antigen vaccine including rApfA, rApxIA to IVA and rTbpB, or with PBS in oil adjuvant, respectively (Table 2). Animals in the last group were neither immunized nor challenged. Two independent immunization and challenge experiments were performed.

**Figure 2:**
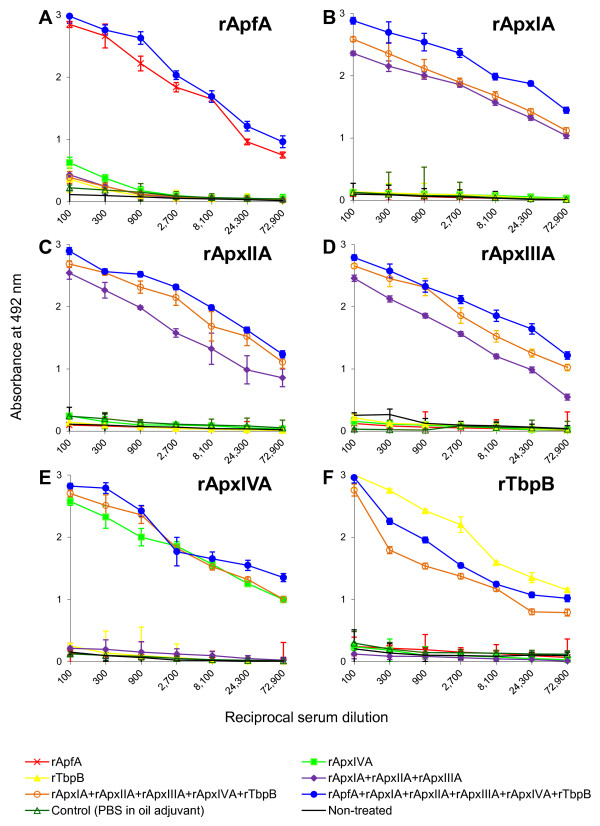
**Titration of porcine post-vaccination sera**. Pigs were immunized with two doses of the indicated vaccines at two week intervals and the post-vaccination sera from all piglets were taken one day before challenge and examined for the anti-rApfA (A), anti-rApxIA (B), anti-rApxIIA (C), anti-rApxIIIA (D), anti-rApxIVA (E) and anti-rTbpB (F) antibody levels using ELISA. Each point represents an average value calculated from results obtained for titration of sera taken from all pigs included in the given vaccination group ± standard deviation.

The post-vaccination sera from all piglets were taken one day before challenge and examined for anti-rApfA, anti-rApxIA to IVA and anti-rTbpB antibody levels using ELISA. As documented in Figure 2, the post-vaccination sera taken from all animals of a given vaccination group exhibited high titers (over 72 900) of antibodies against each of the individual component of the vaccine, but did not contain antibodies against an antigen that was not included in the vaccine. As further documented in Figure 2, no significant levels of rApfA, rApxIA to IVA or rTbpB specific antibodies were detected in sera of animals immunized with PBS in oil adjuvant or in sera of non-treated animals. As a control, preimmune sera were tested for the presence of anti-rApfA, anti-rApxIA to IVA and anti-rTbpB antibodies and were found to be negative (data not shown). Altogether, these results demonstrated that the rApfA, rApxIA to IVA and rTbpB proteins were highly immunogenic and induced specific serum antibodies.

The protective potency of the vaccines was tested in heterologous challenge experiments using an infectious dose of 2 × 10^8^CFU of *A. pleuropneumoniae *serotype 9 strain that was applied via the intranasal route one month after the first immunization. In the control group of animals that received PBS in oil adjuvant, all pigs suffered from severe cough, dyspnea, apathy and fever fluctuating between 40.8-41.6°C within seven days following the challenge with *A. pleuropneumoniae *serotype 9 strain (Table 2). Two out of seven pigs died within 24 h after the challenge and one animal was found dead on the second day after inoculation, while the remaining pigs survived until the end of the study, despite severe clinical signs of porcine pleuropneumonia. Upon necropsy, all pigs in this group showed lung lesions characterized by a hemorrhagic necrotizing pneumonia and fibrinous pleuritis typical of *A. pleuropneumoniae *infection with the mean lung lesion score 2.3 ± 0.7 out of 3 (Table 2). The challenge strain was re-isolated from the lungs of all pigs, confirming the etiology of the lesions (Table 2). These results demonstrated that the challenge with the heterologous *A. pleuropneumoniae *serotype 9 strain caused typical signs of severe porcine pleuropneumonia.

In contrast to animals that received only PBS in oil adjuvant, the pigs vaccinated with rApfA showed considerably milder clinical signs of porcine pleuropneumonia (Table 2). The animals suffered from coughing, increased respiration rate and mild apathy for seven days after the challenge and fever up to 40.8°C. Corresponding to milder clinical signs of pleuropneumonia, also the mean lung lesion score was significantly lower in animals immunized with rApfA (1.3 ± 0.7, *P *< 0.02), when compared to the control (2.3 ± 0.7) (Table 2). The challenge strain was re-isolated from the lungs of seven out of twelve animals (Table 2).

Immunization with rApxIVA or rTbpB alone or with the tri-antigen combination of rApxIA to IIIA, respectively, did not protect the challenged animals against development of clinical signs of the disease, however, none of the pigs died within seven days following the challenge (Table 2). Upon challenge, the animals immunized with rApxIVA or rTbpB alone suffered mainly from cough, abdominal respiration, dyspnea, apathy and fever up to 41.4°C. Upon necropsy, a massive destruction of the lung tissue was observed (Table 2). On the contrary, the pigs vaccinated with the tri-antigen combination of rApxIA to IIIA showed milder clinical signs of porcine pleuropneumonia and lower mean lung lesion score than the animals vaccinated with rApxIVA or rTbpB alone (Table 2). The challenge strain was recovered from the lungs of all pigs vaccinated with rApxIVA or rTbpB alone or with the tri-antigen combination, respectively (Table 2).

All these data demonstrate that rApfA alone provided a partial protection against infection with *A. pleuropneumoniae *that was higher than the protection conferred by the immunization with rApxIVA or rTbpB alone and similar to the protection after vaccination with the tri-antigen combination of rApxIA, rApxIIA and rApxIIIA.

When the purified rApxIA to IVA and rTbpB proteins were combined in a penta-antigen vaccine and used for immunization, a high level of protection against the challenge with *A. pleuropneumoniae *serotype 9 strain was observed (Table 2). Pigs mainly suffered from increased respiration rate, coughing, occasional lying down and mild fever not exceeding 40.3°C. The observed clinical signs had a tendency to diminish over the seven day period after the challenge. The mean lung lesion score was significantly lower in animals immunized with the combined penta-antigen vaccine (1.0 ± 0.6, *P *< 0.02), when compared to the control group (2.3 ± 0.7) (Table 2). The challenge strain was re-isolated from the lungs of six out of seven pigs included in this experimental group (Table 2). These data indicate that the penta-antigen vaccine provided high level of protection against acute disease upon challenge with *A. pleuropneumoniae *.

When this penta-antigen vaccine was supplemented with the purified rApfA and used for immunization, the combined hexa-antigen vaccine containing rApfA, rApxIA to IVA and rTbpB, respectively, conferred on animals a high level of protection against all clinical signs accompanying acute infection (Table 2). Pigs showed only sporadic coughing and occasional lying down for the first three days and presented mild fever about 40.1°C during the first two days after the challenge. After the period of three days, none of the animals showed any clinical signs of the disease or fever. Following necropsy, none or only minor pathological changes were observed in the lungs of these animals and the mean lung lesion score was 0.3 ± 0.5 (Table 2), which was fully in line with the absence of signs. The challenge strain was isolated from the lungs of only two out of the seven challenged animals (Table 2). These results demonstrate that rApfA further improved the vaccine potency of the currently used antigens and that the immunization with the combined hexa-antigen vaccine consisting of rApfA, rApxIA to IVA and rTbpB enabled a high level of protection of pigs against pleuropneumonia. Moreover, rApfA used alone or in combination with other antigens significantly decreased the number of animals colonized by the heterologous challenge strain of *A. pleuropneumoniae *. Hence, ApfA should be included in a new generation of subunit vaccines against porcine pleuropneumonia, providing not only a complete protection of pigs against the clinical signs of the acute disease, but also limiting lung colonization by *A. pleuropneumoniae *.

## Discussion

To develop more effective subunit vaccines against porcine pleuropneumonia, the identification, production and testing of novel *A. pleuropneumoniae *protein antigens are the objectives of current research. We show here that the type IV fimbrial subunit protein ApfA should be considered as a promising vaccine candidate.

We demonstrate that vaccination of pigs with the recombinant ApfA protein alone provided limited protection against heterologous challenge with *A. pleuropneumoniae *. This result was in agreement with previous reports showing that single antigen vaccines offer only partial or no protection against pleuropneumonia. For instance, when ApxIIA and TbpB proteins expressed in *E. coli *were used for vaccination separately, all pigs developed a strong humoral immune response and upon challenge with a homologous *A. pleuropneumoniae *serotype 7 strain, all immunized animals were less affected by the pleuropneumonia and demonstrated significantly lower mortality than the controls [53]. However, protection was serotype-specific, since no cross-protection was detected, when the animals were challenged with an *A. pleuropneumoniae *serotype 1 strain [53]. Recently, the N-terminal portion of ApxIVA produced in *E. coli *was used for vaccination and the challenged pigs showed similar severe respiratory signs and severe lung lesions as the animals in the control group, despite high antibody titers against the N-terminus of ApxIVA [49]. We obtained similar results showing that the vaccination of pigs with full-length rApxIVA or rTbpB alone protected animals against death upon heterologous challenge with *A. pleuropneumoniae *serotype 9 strain, but did not protect them against development of clinical signs of the disease and massive destruction of the lung tissue, despite high titers of specific antibodies. In contrast, the pigs immunized with the recombinant ApfA alone showed milder clinical signs of porcine pleuropneumonia and reduced destruction of the lung tissue than the animals vaccinated with rApxIVA or rTbpB alone.

Since single antigens often provide only limited protection against porcine pleuropneumonia, multi-antigen subunit vaccines were used for immunization. For instance, pigs receiving a mixture of the recombinant ApxIIA and TbpB proteins had a tendency to recover faster than animals that were vaccinated with only one antigen [53]. Similarly, the N-terminal portion of ApxIVA improved protective immunity conferred by the subunit vaccine containing recombinant ApxIA, ApxIIA, ApxIIIA and the 42-kDa outer membrane protein [49]. Therefore, we also tested whether recombinant ApfA would improve protection upon its addition to a multi-antigen subunit vaccine. Our data demonstrate that the immunization of pigs with the combined hexa-antigen vaccine consisting of rApfA, rApxIA to IVA and rTbpB provided protective immunity against pleuropneumonia that was significantly higher than that acquired upon vaccination with rApfA, rApxIVA or rTbpB alone, with the tri-antigen combination of rApxIA, rApxIIA and rApxIIIA or even with the penta-antigen subunit vaccine composed of rApxIA to IVA and rTbpB. Thus, rApfA improved the vaccination potential of currently used antigens. Moreover, immunization of pigs with the combined hexa-antigen vaccine provided almost complete protection of animals against the heterologous challenge with *A. pleuropneumoniae *serotype 9 strain.

Recently, the contribution of recombinant ApfA to the protective immunity of a subunit vaccine comprising rApxIA, rApxIIA, rApxIIIA and rOMP was tested in mice [71]. It has been shown that the antibody titers against rApxIA, rApxIIA, rApxIIIA and rOMP were significantly lower in sera of mice immunized with the vaccine containing rApfA, rApxIA to IIIA and rOMP than the titers in sera of mice immunized with the vaccine comprising only rApxIA to IIIA and rOMP. In agreement, upon challenge with *A. pleuropneumoniae*, mice immunized with the vaccine containing rApfA, rApxIA to IIIA and rOMP had the lower survival rate and more severe lung lesions than the animals immunized with the vaccine lacking rApfA. Thus, it has been suggested that vaccination with rApfA may impair immunity of mice [71], similarly as described for the outer membrane protein PalA of *A. pleuropneumoniae *in pigs [68]. Our immunization and challenge experiments in pigs, however, do not support the observation achieved with rApfA in mice. As documented in Figure 2, we clearly show that the post-vaccination sera of pigs immunized with the hexa-antigen vaccine containing rApfA, rApxIA to IVA and rTbpB exhibited higher or comparable levels of rApxIA, rApxIIA or rApxIIIA specific antibodies than the sera of animals immunized with the penta-antigen vaccine comprising only rApxIA to IVA and rTbpB. In addition, the hexa-antigen vaccine conferred on pigs a significantly better protection against pleuropneumonia than did the penta-antigen vaccine lacking rApfA (Table 2).

Type IV fimbriae are known to play a key role in virulence of many species of Gram-negative bacteria and therefore type IV fimbrial subunit proteins have been tested as subunit vaccines for the prevention of several diseases. For instance, a highly purified fimbriae prepared from cells of *Dichelobacter nodosus *provided a high level of protection against ovine foot-rot upon homologous challenge and lower levels of protection against challenge with strains of different serogroups [72]. Similarly, type IV fimbriae isolated and purified from different strains of *Moraxella bovis *effectively protected cattle against experimentally induced infectious bovine keratoconjunctivitis upon homologous challenge, but provided only partial protection against challenge with a strain of different serogroup [73]. Moreover, a change in pilus antigenicity by pilin gene inversion enabling bacterial escape from protective immunity was suggested to occur in response to the presence of pilin-specific antibodies upon vaccination with purified fimbriae of *M. bovis *[74]. The antigenic variation of the pilin subunit has also been an important obstacle to the development of an effective pilus-based vaccine for *Neisseria gonorrhoeae *[75].

We demonstrate here that despite high antibody titers against rApfA, vaccination of pigs with rApfA alone provided only partial protection against heterologous challenge with *A. pleuropneumoniae *. However, the *apfA *alleles were found to be highly conserved and present in a single copy in the genomes of different *A. pleuropneumoniae *serotype strains [18]. Therefore, the observed partial protection of immunized animals against pleuropneumonia was most probably not due to the low level of the ApfA sequence homology or the pilin antigenic variation in the challenge strain. A more plausible explanation is that some additional components to type IV fimbriae are involved in adherence of *A. pleuropneumoniae *to alveolar epithelial cells, which is an important initial step in the pathogenesis of porcine pleuropneumoniae [20,76]. Indeed, *A. pleuropneumoniae *was shown to bind to phosphatidylethanolamine of the host cells through O-antigen of its cell-wall lipopolysaccharides [77]. Moreover, a 55-kDa outer membrane protein (OMP) was shown to play a role in the adherence of *A. pleuropneumoniae *to alveolar epithelial cells in culture [78]. Thus, a multiple-step binding process has been suggested, in which *A. pleuropneumoniae *first uses low-affinity binding between O-antigen and phospholipids or short glycolipids and then relies on the core oligosaccharide of LPS and/or surface proteins, such as 55-kDa OMP and type IV fimbriae, to interact more avidly with other larger lipidic and/or proteinaceous receptors on host cells [77]. The use of multiple parallel adhesion mechanisms by *A. pleuropneumoniae *and involvement of additional virulence factors would, hence, provide a likely explanation for the observed only partial protective efficacy of the vaccine based solely on ApfA, which was not able to completely prevent the colonization of the lungs of pigs by the challenge strain of *A. pleuropneumoniae *. Conversely, the reduction of the number of pigs colonized by the challenge strain up to 29% upon immunization with the ApfA-based vaccines suggests that ApfA-specific antibodies might markedly inhibit the pilus-mediated binding of *A. pleuropneumoniae *to host cells, despite the existence of other adherence mechanisms.

In conclusion, our results demonstrate that immunization of pigs with the type IV fimbrial subunit protein ApfA induced specific serum antibody response that reduced clinical signs of the acute disease upon heterologous challenge with *A. pleuropneumoniae *and significantly increased the potency of the currently used protective antigens. Thus, ApfA could be a candidate to be included in a next generation of subunit vaccines against porcine pleuropneumonia.

## Competing interests

The authors declare that they have no competing interests.

## Authors' contributions

LS participated in the design of the study, performed vector construction, protein expression, purification and analysis, carried out ELISA experiments, analyzed data and drafted the manuscript. JN participated in the design of the study and in the development of ELISA protocols, performed immunizations and challenge experiments in pigs and collected and analyzed data. VV conceived of the study, participated in its design and coordination. PS participated in the design of the study and edited the manuscript. RO participated in the design of the study, in the development of ELISA protocols and protein expression and purification methods and edited the manuscript. All authors read and approved the final manuscript.
